# In-vitro NET-osis induced by COVID-19 sera is associated to severe clinical course in not vaccinated patients and immune-dysregulation in breakthrough infection

**DOI:** 10.1038/s41598-022-11157-0

**Published:** 2022-05-04

**Authors:** Alessandra Romano, Nunziatina Laura Parrinello, Martina Barchitta, Rosy Manuele, Fabrizio Puglisi, Andrea Maugeri, Alessandro Barbato, Anna Maria Triolo, Cesarina Giallongo, Daniele Tibullo, Lucia La Ferla, Ciro Botta, Sergio Siragusa, Carmelo Iacobello, Arturo Montineri, Giovanni Li Volti, Antonella Agodi, Giuseppe Alberto Palumbo, Francesco Di Raimondo

**Affiliations:** 1 Division of Hematology, Azienda Policlinico-Rodolico San Marco, Catania, Italy; 2 Dipartimento di Chirurgia Generale e Specialità Medico Chirurgiche, University of Catania, Catania, Italy; 3 Department of Medical and Surgical Sciences and Advanced Technologies “GF Ingrassia”, University of Catania, 95123 Catania, Italy; 4 U.O.C. di Malattie Infettive, Azienda Policlinico-Rodolico San Marco, Catania, Italy; 5 Dipartimento di Scienze Biomediche e Biotecnologiche, University of Catania, Catania, Italy; 6 U.O.C. di Malattie Infettive, Azienda Cannizzaro, Catania, Italy; 7 Division of Hematology, Università degli Studi di Palermo, Palermo, Italy

**Keywords:** Antimicrobial responses, Cytokines, Viral infection

## Abstract

Since neutrophil extracellular traps formation (NET-osis) can be assessed indirectly by treating healthy neutrophils with blood-derived fluids from patients and then measuring the NETs response, we designed a pilot study to convey high-dimensional cytometry of peripheral blood immune cells and cytokines, combined with clinical features, to understand if NET-osis assessment could be included in the immune risk profiling to early prediction of clinical patterns, disease severity, and viral clearance at 28 days in COVID-19 patients. Immune cells composition of peripheral blood, cytokines concentration and in-vitro NETosis were detected in peripheral blood of 41 consecutive COVID-19 inpatients, including 21 mild breakthrough infections compared to 20 healthy donors, matched for sex and age. Major immune dysregulation in peripheral blood in not-vaccinated COVID-19 patients compared to healthy subjects included: a significant reduction of percentage of unswitched memory B-cells and transitional B-cells; loss of naïve CD3^+^CD4^+^CD45RA^+^ and CD3^+^CD8^+^CD45RA^+^ cells, increase of IL-1β, IL-17A and IFN-γ. Myeloid compartment was affected as well, due to the increase of classical (CD14^++^CD16^−^) and intermediate (CD14^++^CD16^+^) monocytes, overexpressing the activation marker CD64, negatively associated to the absolute counts of CD8+ CD45R0+ cells, IFN-γ and IL-6, and expansion of monocytic-like myeloid derived suppressor cells. In not-vaccinated patients who achieved viral clearance by 28 days we found at hospital admission lower absolute counts of effector cells, namely CD8^+^T cells, CD4^+^ T-cells and CD4^+^CD45RO^+^ T cells. Percentage of in-vitro NET-osis induced by patients’ sera and NET-osis density were progressively higher in moderate and severe COVID-19 patients than in mild disease and controls. The percentage of in-vitro induced NET-osis was positively associated to circulating cytokines IL-1β, IFN-γ and IL-6. In breakthrough COVID-19 infections, characterized by mild clinical course, we observed increased percentage of in-vitro NET-osis, higher CD4+ CD45RO+ and CD8+ CD45RO+ T cells healthy or mild-COVID-19 not-vaccinated patients, reduced by 24 h of treatment with ACE inhibitor ramipril. Taken together our data highlight the role of NETs in orchestrating the complex immune response to SARS-COV-2, that should be considered in a multi-target approach for COVID-19 treatment.

## Introduction

The outcome of infection with the severe acute respiratory syndrome coronavirus 2 (SARS-CoV-2) varies broadly. In most cases, COVID-19 symptoms are moderate^1,2^ or absent at all^3^. Around 15% of patients can progress to severe pneumonia and about 5% eventually develop acute respiratory distress syndrome, septic shock and/or multiple organ failure^4^. The severity of symptoms varies by age and strength and duration of the immune responses to SARS-CoV-2^5^. However, despite intensive efforts in the field, it is still to clarify why the response to infection varies so much from person to person and which immunopathological mechanisms lead to severe disease.

Clinical course and disease severity are strongly associated to weaker immune response to the virus. However, severe clinical manifestations may be caused by hyperactivated and misdirected immune responses, as high levels of IL-6 triggering cytokine storm in the absence of appropriate type I and III interferon (IFN) responses^6^. Severe SARS-CoV-2 is characterized by two unique signatures of immune dysregulation, one with normal or high cytokine production capacity and increased circulating cytokines (especially IL-6) and one by defects in the myeloid and lymphoid function associated with IL-6-mediated decrease in HLA-DR expression^7^, with several intermediate states.

In vitro, SARS-CoV-2 can induce a functional specialization of dendritic cells subsets, leading to high levels of interferon-α, interferon-λ1, IL-6, IP-10 and IL-8, to orchestrate and propagate first the innate and then the adaptive immune response. Thus, the perturbed immune parameters observed in critically ill COVID-19 patients are probably acquired during disease evolution through secondary events^8^. The viral recognition upon infection triggers the inflammasome recruitment lee^9,10^ with consequent release of key pro-inflammatory cytokines IL-1ß and IL-18 to trigger the pyroptotic cell death. As a result of pyroptosis, the enzyme lactate dehydrogenase LDH is released and inflammation and coagulopathy triggered^10^. This could explain why we found a positive correlation between LDH, IL-6, IL-1ß, C-RP and disease severity.

Neutrophil Extracellular Traps (NETs) are web-like structures composed of decondensed chromatin in complex with over 30 different neutrophil proteins that can capture, neutralize, and kill a variety of microbes, including bacteria, fungi, viruses, and protozoan parasites. Recently, NETs have been described to regulate B cell function in the spleen and to play a role in various sterile diseases, such as autoinflammation, cancer and autoimmune diseases. Myeloid impairment due to increased generation of NET^11–13^, recruitment of emergency hematopoiesis, with release of pre- and -pro-neutrophils^13^ and decrease of non-classical monocytes is commonly observed in severe infections progressing through sepsis in which hyper-inflammatory states lead to immune suppression. Neutrophil extracellular traps (NETs) formation, also called NETosis, can be induced by the lung epithelium damage and it has been implied in the pathogenesis of COVID-19 severity^14^. However, standardized conditions are required for evaluation of neutrophil function, that could explain the variation in study design and results of NETosis in COVID-19 patients.

Since NET-osis can be assessed indirectly by treating healthy neutrophils with blood-derived fluids from patients and then measuring the NETs response^15^, we designed a pilot study to convey high-dimensional cytometry of peripheral blood immune cells and cytokines, combined with clinical features, to understand if NET-osis assessment could be included in the immune risk profiling to early prediction of clinical patterns, disease severity, and viral clearance at 28 days.

## Results

### Demographics and clinical characteristics of mild, moderate, and severe COVID-19^+^ individuals

Patients were categorized as fully vaccinated at the time of COVID-19 when two doses of vaccine BNT162b2 had been administered and diagnosis of COVID-19 was recorded > 4 weeks from the last dose, thus identifying the breakthrough infections after COVID-19 vaccination. Unvaccinated patients were defined as having no known prior exposure to COVID-19 vaccination before COVID-19 diagnosis. Thus, 21 outpatients with breakthrough infections after COVID-19 vaccination were included in the study. All of them developed only mild disease and did not require hospitalization. 20 inpatients were admitted at the two COVID Units for symptomatic COVID-19 patients, including 5 (25%) mild-, 7 (35%) moderate—and 8 (40%) patients with severe disease. Control cohort consisted of 20 healthy donors (HDs), matched for sex and age (Supplementary Fig. 1). According to the available literature, severe disease was defined as requiring oxygen at a flow rate higher than 6 L/min or by an advanced oxygen delivery device including invasive mechanical ventilation, noninvasive ventilation, or high flow nasal cannula because more than 6 L is considered high flow oxygen^16^, associated or not to tachypnea (more than 30 breaths per minute), hypoxemia (oxygen saturation, ≤ 93%; ratio of partial pressure of arterial oxygen to fraction of inspired oxygen, < 300), and presence of lung infiltrates. Moderate disease was defined by the presence of clinical or radiographic evidence of lower respiratory tract disease but with a blood oxygen saturation of 94% or higher while the patient was breathing ambient air^17^.

Peripheral blood was collected by 24 h from hospital admission or confirmed SARS-CoV-2 infection by positive PCR test. Median follow up after enrollment was 35 days (range, 20 to 43) since blood draw to two consecutive negative PCR tests. General demographics and clinical characteristics are shown in Table 1.

**Table 1:** Descriptive statistics of the study population at baseline of their COVID-19 diagnosis.

Characteristics	All patients (N = 20)	Mild COVID19 (N = 5)	Moderate COVID19 (N = 7)	Severe COVID19 (N = 8)	p-value^a^	Breakthrough COVID19 (N = 21)
Median age, years (range)	62 (41–82)	49 (41–77)	60 (43–77)	67 (43–82)	0.58	46 (29–63)
Males, N (%)	11 (55)	1 (20)	4 (57)	6 (75)	NA	10 ()
Two or more comorbidities at admission, N (%)	18 (90)	3 (60)	7 (100)	8 (100)	NA	0 (0)
Diabetes	3 (15)	0 (0)	1 (14)	2 (25)	NA	0 (0)
Hypertension	13 (65)	3 (20)	4 (57)	6 (75)	NA	3 ()
Chronic obstructive pulmonary disease	7 (35)	1 (20)	2 (29)	4 (50)	NA	2 ()
Hyperlipidemia	13 (65)	2 (40)	5 (71)	6 (75)	NA	4 ()
Pre-admission administration of drugs for comorbidities	15 (75)	3 (60)	4 (57)	8 (100)	0.82	0 (0)
**Hemochrome parameters, median (range)**						
Haemoglobin, g/dL	12.1 (10.4–15.1)	13.4 (12.7–15.1)	12.1 (10.4–12.6)	11.9 (10.4–12.3)	***0.02***	14.3 (9.4–15.6)
Absolute white blood cells × 10^6^/mmc	6.3 (3.6–8.2)	6.6 (5.5–7.0)	4.6 (3.6–6.9)	6.7 (5.4–8.2)	0.19	6.6 (3.4–12.3)
Absolute neutrophils × 10^6^/mmc	3.6 (2.1–5.1)	4.2 (3.8–5.1)	3.6 (3.3–4.2)	2.7 (2.1–3.1)	0.13	4.2 (2.8–6.1)
Absolute lymphocytes × 10^6^/mmc	1.4 (0.8–2.2)	1.7 (1.4–2.2)	1.5 (0.9–2.2)	1.2 (0.8–1.3)	0.83	1.8 (0.6–2.8)
Absolute monocytes × 10^6^/mmc	0.5 (0.3–0.8)	0.4 (0.3–0.5)	0.5 (0.3–0.6)	0.5 (0.4–0.8)	0.96	0.4 (0.2–0.6)
Absolute platelets × 10^9^/mmc	228 (179–281)	226 (216–257)	185 (152–260)	223 (180–348)	0.92	242 (89–410)
Median LDH, UI/L (range)	253 (136–512)	156 (136–217)	239 (159–300)	316 (270–512)	***0.03***	156 (120–332)
Median reactive C-protein, UI/L (range)	22.1 (1.6–46.7)	2.0 (1.7–3.8)	9.1 (1.4–22.3)	32.1 (8.1–46.2)	***0.01***	2.3 (1.8–3.9)
Median IgA, g/dL (range)	285 (210–687)	296 (245–360)	306 (210–368)	280 (200–687)	0.86	290 (240–360)
Median IgM, g/dL (range)	72 (60–123)	75 (72–106)	68 (70–123)	66 (62–119)	0.62	96 (85–132)
Median IgG, g/dL (range)	1040 (930–1476)	1042 (956–1380)	1060 (930–1376)	1011 (940–1476)	0.66	1086 (870–1400)
Median d-dimer, ng/mL (range)	445 (88–792)	103 (88–138)	371 (149–524)	503 (304–792)	***0.04***	30 (8–16)

^a^Based on ANOVA test for the comparison of findings in COVID-19 patients; p-values < 0.05 are indicated in bold font. Results are reported as median and interquartile (IQ) range.

In the group of not vaccinated patients, the median ages in the mild, moderate, and severe COVID-19 + groups were 49, 60 and 67 years old, respectively, and were not significantly different (p = 0.08). Most individuals in the severe group were male (N = 6, 75%), whereas 57% and 20% were male respectively in the moderate and in the mild disease groups. The median number of days since onset of symptoms to hospital admission was 7 (range 5–10). 90% of patients were treated with two or more drugs for comorbidities. Patients with severe COVID-19 had high incidence of underlying pulmonary disease and chronic obstructive pulmonary disease (COPD, 50%), hypertension (75%), hyperlipidemia (75%) and uncontrolled diabetes (25%). One patient with positive anamnesis for acute disseminated encephalomyelitis (ADEM) and one affected by multiple sclerosis received high-doses immunoglobulins for their comorbidity. Hypertension and hyperlipidemia were the most frequent comorbidities in mild and moderate COVID-19. According to the inclusion criteria, no patients had active, uncontrolled malignancy or autoimmune disease or received high-doses steroids.

As part of clinical care, we measured differential count blood, D-dimer, lactate dehydrogenase, and C-reactive protein levels (Table 1). Median levels of D-dimer at the time of blood draw were 503 ng/ml in severe, 371 ng/ml in moderate and 103 ng/mL in mild COVID-19 patients (p = 0.04). Similarly, patients with severe disease had higher counts of LDH and reactive C protein than patients referred for moderate and mild disease (respectively 316 vs 239 vs156 UI/L, p = 0.03 and 32.1 vs 9.1 vs 2.0, p = 0.01). Differential counts of white blood cells were similar across severity groups (Table 1).

### Cytokine storm at hospital admission of symptomatic not-vaccinated COVID-19 patients

To assess the general landscape of immune perturbation in different clinical settings of COVID-19, we contemporary measured the concentration in peripheral blood of six cytokines involved in the inflammation orchestrating: IL-6, IL-1β, IL-17A, TGF-β, TNF-α and IFN-γ (Table 2).

**Table 2:** Concentration in peripheral blood of cytokines involved in the immune response in patients recently admitted at COVID-19 Units.

	Healthy (N = 20)	COVID-19			p-value^a^
		Mild (N = 5)	Moderate (N = 7)	Severe (N = 8)	
**IL1-β**					
Mean (pg/mL)	45.9	82.6	98.8	93.3	0.12
SD	28.4	8.9	31.8	28.3	
**TNF-α**					
Mean (pg/mL)	6.9	7.7	8.7	12.0	0.09
SD	0.3	0.5	0.8	0.9	
**TGF-β**					
Mean (pg/mL)	8274	6982	9501	19,811	0.32
SD	1790	2504	1403	2632	
**IFN-γ**					
Mean (pg/mL)	8.8	190.6	238.3	264.8	*0.002*
SD	0.9	0.4	37.3	55.8	
**IL-6**					
Mean (pg/mL)	0.9	1.8	27.1	196.3	< *0.001*
SD	0.2	0.1	8.3	80.3	
**IL-17A**					
Mean (pg/mL)	11.5	71.7	89.1	127.1	*0.001*
SD	0.65	6.2	0.7	9.4	

Significant values are in italics.

^a^ANOVA test for the comparison of cytokine in COVID-19 patients, results are reported as means and standard deviations.

IL-1β was doubled in COVID-19 patients compared to healthy subjects, with a positive trend due to severity, in lack of statistical significance. Patients with mild and moderate COVID-19 had similar levels of TNF-α than healthy subjects. TNF-α was significantly higher in severe COVID-19 patients than healthy subjects (12.0 ± 0.9 vs 6.9 ± 0.3 pg/mL, p = 0.003, t-test). Similarly, TGF-β was significantly higher in severe COVID-19 patients than healthy subjects (19,811 ± 2632 vs 8274 ± 1790 pg/mL, p = 0.003, t-test), despite no significance in the ANOVA test. IFN-γ was higher in patients compared to healthy subjects, with a significant increase from mild, moderate, and severe disease (respectively, 190.6 ± 0.4 vs 238.3 ± 37.3 vs 264.8.1 ± 55.8 pg/mL, p = 0.002, ANOVA-test). As previously reported, IL-6 was higher in severe and moderate COVID-19 patients than healthy and mild COVID-19 subjects (p < 0.001). Similarly, IL-17A was higher in patients compared to healthy subjects, with a significant increase from mild, moderate, and severe disease (respectively, 71.7 ± 6.2 vs 89.1 ± 0.7 vs 127.1 ± 9.4 ng/mL, p = 0.001, ANOVA-test, (Table 2).

**Figure 1:**
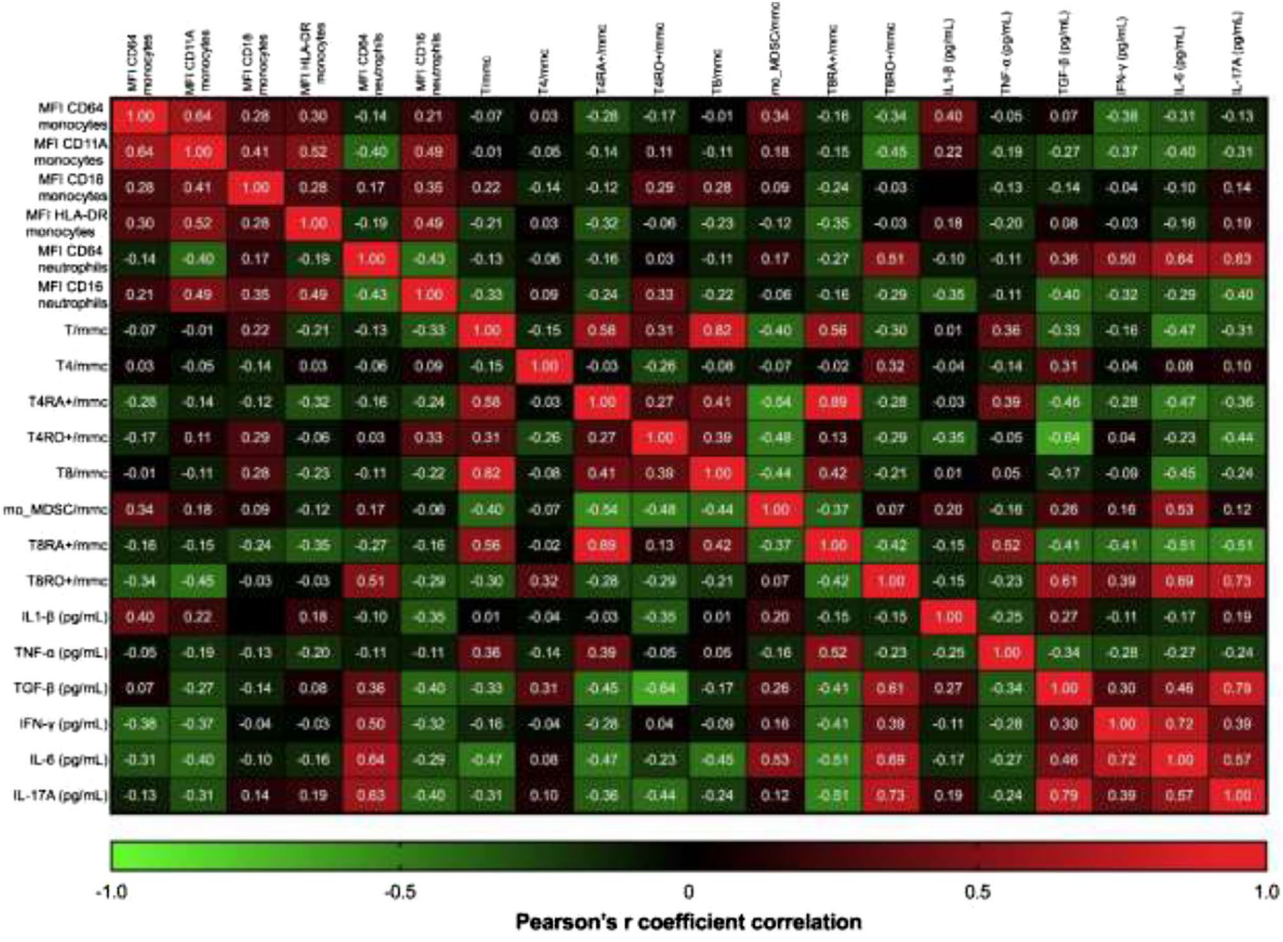
The correlation matrix and heatmap of Pearson’s r correlation coefficients among the multiple variables included in the analysis of COVID-19 patients. p-values of each comparison have been separately reported in Supplementary Table 1.

Independently from disease severity, IL-6 was positively associated with levels of TGF-β and IFN-γ (r-square respectively 0.46, p = 0.0021 and 0.72, p = 0.003), while IFN-γ was positively associated with IL-17A levels (r-square 0.39, p < 0.0001) as shown in Fig. 1.

### Immune perturbation in severe not-vaccinated COVID-19 patients

**Figure 2:**
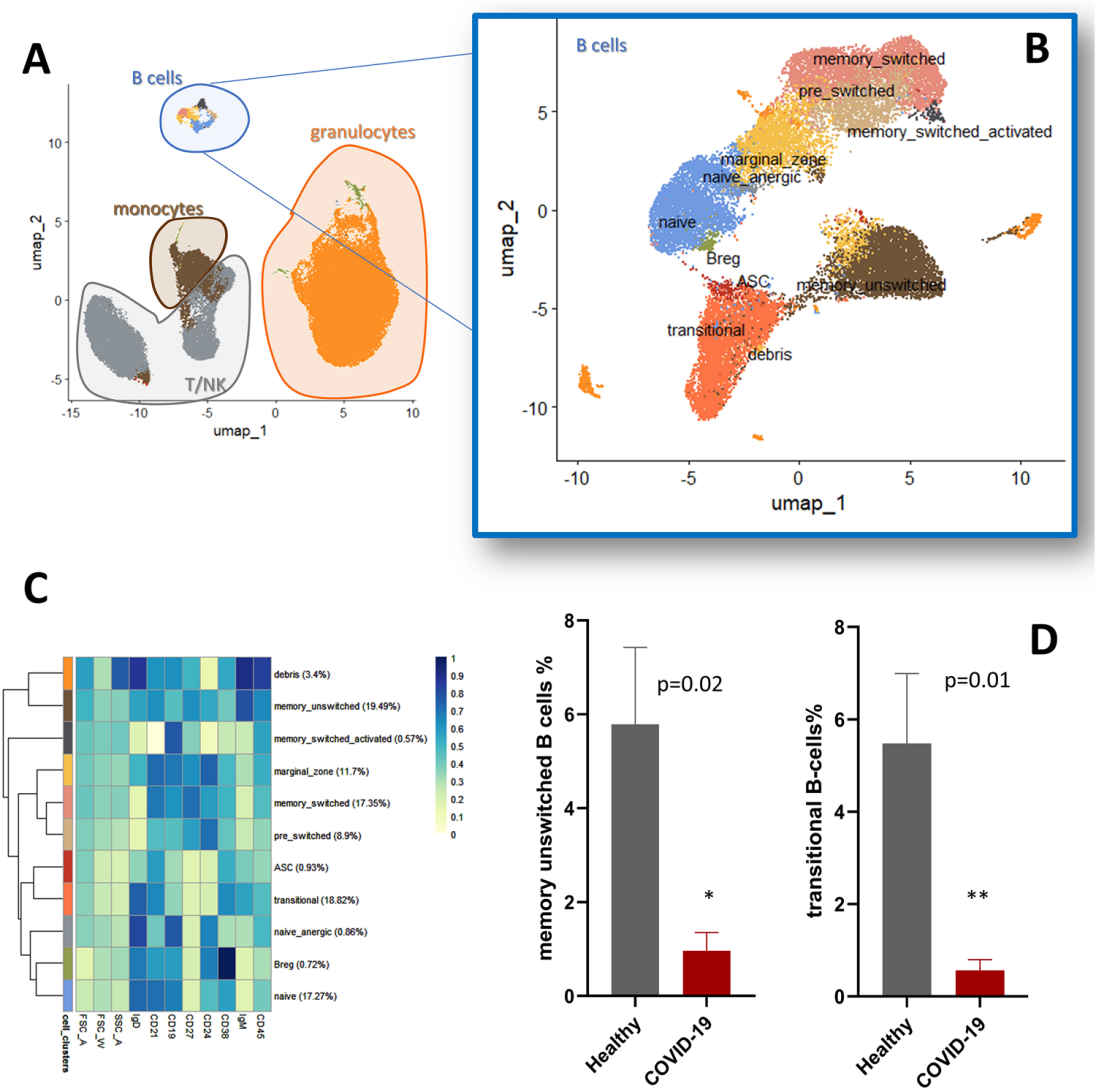
Dysregulation of B-cells composition in peripheral blood of COVID-19 patients. Cell composition and expression differences between COVID-19 and healthy peripheral blood. UMAP of CD45+ leukocytes **(A)**. Automatic prediction identifies different subsets in the B-cell -compartment **(B)**. MFI expression changes in B-cells (rows: identified sub-populations; columns: antigen of interest **(C)**. Memory-switched B cells and transitional B cells were lower in COVID-19 patients (red bars) than in healthy subjects (grey bars) **(D)**. *p < 0.005, **p < 0.001, t-test.

**Figure 3:**
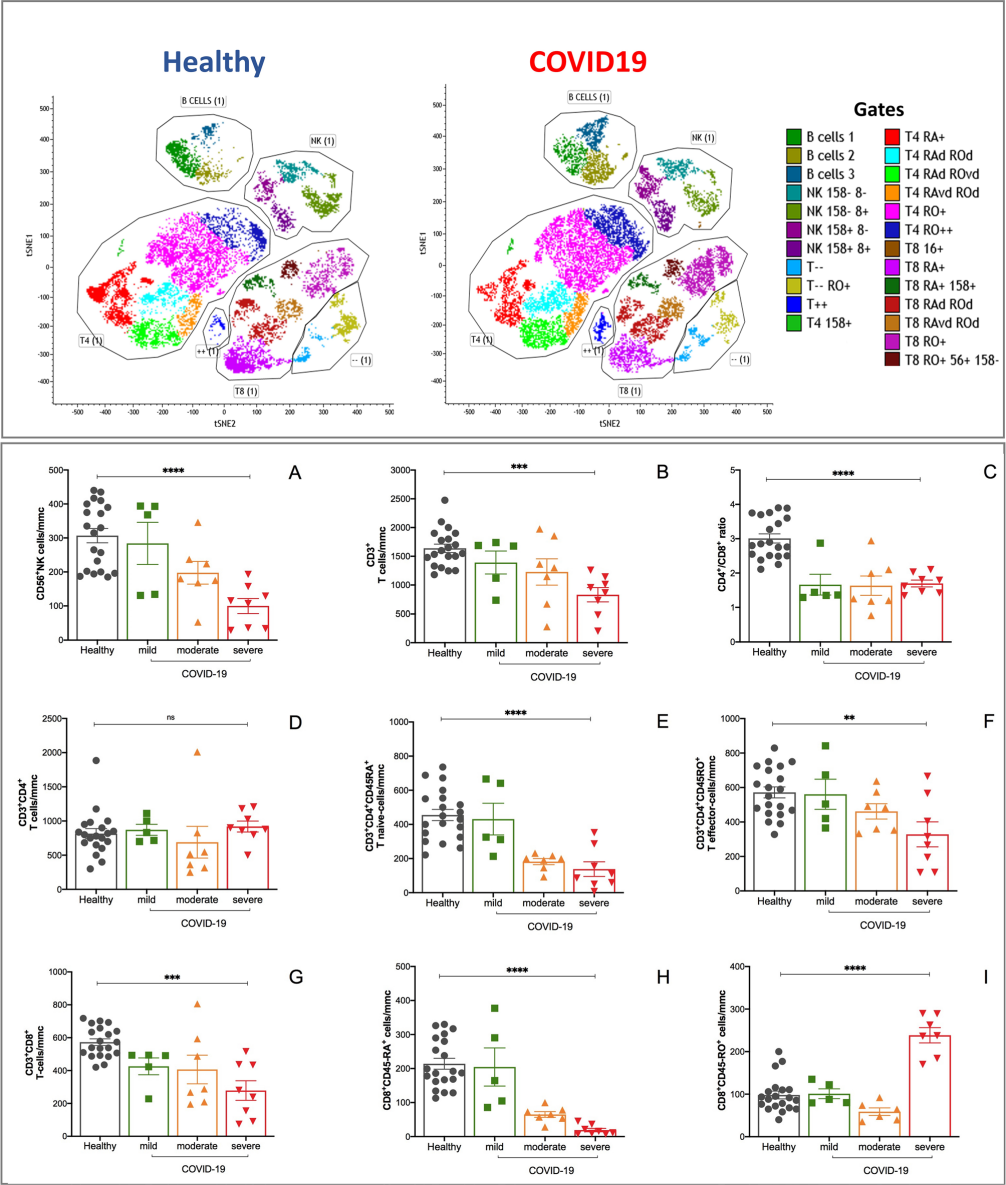
Dysregulation of T-cells composition in peripheral blood of COVID-19 patients. Application of T-distributed stochastic neighbor embedding (t-SNE): clusters were named based on the cluster-specific antigen expression patterns in healthy subjects **(A)** and COVID-19 patients **(B)**. Ratio between absolute counts of CD4^+^/CD8^+^ T-cells **(C)**, CD4^+^CD45RA^+^/CD8^+^CD45RA^+^/T-cells **(D)** and CD4^+^CD45RO^+^/CD8^+^CD45RO^+^/T-cells **(E)**, CD4^+^T-cells **(F)**, CD4^+^CD45RA^+^ **(G)**, CD4^+^CD45RO^+^ **(H)**, CD8^+^T-cells **(I)**, CD8^+^CD45RA^+^ **(L)**, CD8^+^CD45RO^+^ **(M)** are shown. Color code: grey, healthy subjects; green, mild COVID-19; orange, moderate COVID-10, red, severe COVID-19. COVID-19 severity was defined according to qCSI, based on respiratory rate, pulse oximetry and oxygen flow rate. Stars denote significance according to ANOVA test among the three COVID groups (mild, moderate, severe), *p < 0.005, **p < 0.001.

To further investigate the general landscape of immune phenotype in different clinical settings of COVID-19, we measured the frequencies of circulating immune subsets in mild, moderate, and severe COVID-19^+ ^inpatients compared with HDs. We used t-SNE and UMAP analysis to capture the multi-dimensional geometric relationships between single cells (Figs. 2 and 3) to identify immune perturbation in severe COVID-19 in the following compartments: B-cells, NK cells, T-cells, monocytes, and neutrophils. A small fraction of putative dendritic cells was left unidentified, due to lack of specific markers for further characterization.

### Pattern of serum immunoglobulins and impaired compartmentalization of B-cells in not-vaccinated COVID-19 patients

At blood collection, in all patients we could detect IgM (but no IgG) antibodies against the virus and normal amounts of different classes of immunoglobulins (Table 1). We did not observe a difference in total CD19^+^ B cell frequency between COVID-19 and healthy subjects (data not shown). Memory B cell frequencies ranged widely, in both COVID-19 and healthy subjects, potentially due to the minimal exclusion criteria used to select healthy volunteers. Despite this limitation, statistical analysis of unbiasedly identified populations (see UMAP in Fig. 2A–C) revealed a significant reduction of percentage of unswitched memory B-cells and transitional B-cells in COVID-19 patients compared to healthy subjects (respectively 5.8 ± 1.6 vs 0.9 ± 0.4%, p = 0.02, and 5.4 ± 1.5 vs 0.6 ± 0.2%, p = 0.01, Fig. 2D).

### Severity-dependent alterations of NK- and T-cells in not-vaccinated COVID-19 patients

Consistent with previous reports, we observed a relative decrease in the percentages of all lymphocyte subsets. T-distributed stochastic neighbor embedding (t-SNE) analysis showed reduction of T cell numbers, including total T cells, CD4^+^ and CD8^+^ T cells in COVID-19 patients compared to healthy subjects, matched for age and sex (Fig. 3, upper panel). T cell loss associated to severe COVID-19 disease was mainly due to loss of naïve CD3^+^CD4^+^CD45RA^+^ cells and naïve CD3^+^CD8^+^CD45RA^+^ cells. In severe COVID-19 the ratio between naïve CD4+ and CD8+ cells was weakly increased, without achieving a significant difference; on the opposite, the ratio between effector CD4+ and CD8+ cells was progressively reduced from mild through moderate and severe disease (p = 0.01, ANOVA-test), due to the combined reduction of both CD4+ and CD8+ cells in severe disease, as shown in Fig. 3A–I.

The absolute count of effector CD8^+^CD45RO^+^ cells was positively associated to the amount of TGF-β (r-square 0.61, p = 0.006), IL-17A (r-square 0.73, p = 0.003), IL-6 (r-square 0.69, p = 0.003) and IFN-γ (r-square 0.51, p = 0.007), as shown in Fig. 1.

Despite no changes in the percentages, absolute counts of NK cells were progressively reduced from mild through moderate and severe COVID-19 patients (respectively, 284 ± 62 vs 177 ± 35 vs 109 ± 23 cells/mmc, p = 0.009, ANOVA-test). Consistent with previous reports we observed a marked decrease in the frequencies of both CD56^bright^CD16^−^ and CD56^dim^CD16^+^ NK cells in severe COVID-19 versus HDs (data not shown). There were no significant changes in the median fluorescence intensity of activation marker CD158 among healthy and COVID-19 subjects (data not shown), which was associated to the amount of IL-6 (r-square = 0.61, p = 0.004) and TGF-β (r-square = 0.46, p = 0.004), but not of IL-1β, IL-17A TNF-α or IFN-γ evaluated at hospital admission (data not shown).

### Severity-dependent alterations of the monocyte compartment in not-vaccinated COVID-19 patients

**Figure 4:**
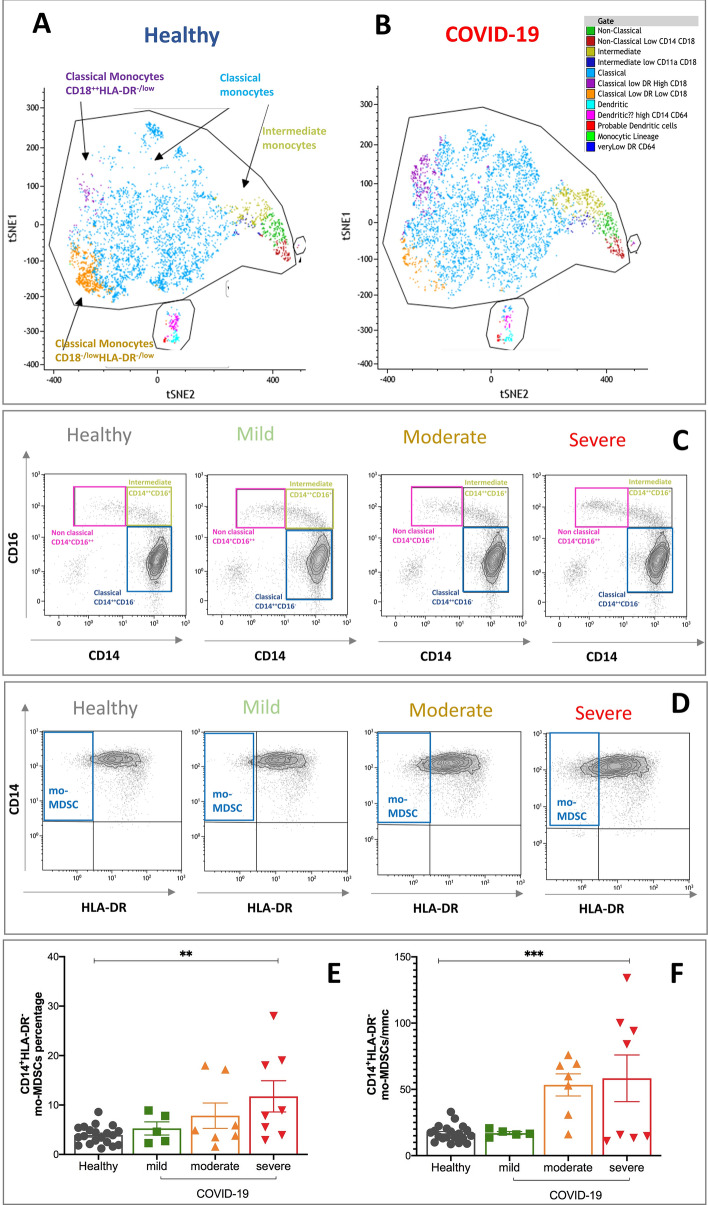
Dysregulation of myeloid cells in peripheral blood of COVID-19 patients. Application of T-distributed stochastic neighbor embedding (t-SNE). Clusters were named based on the cluster-specific antigen expression patterns in healthy subjects **(A)** and COVID-19 patients **(B)**. Example of gating strategy based on CD14 and CD16 expression in circulating monocytes to identify non-classical, intermediate and classical monocytes in healthy and COVID-19 patients **(C)**. Detection of percentage and absolute counts of monocyte-like myeloid derived suppressor cells identified as CD14^+^-HLA-DR^-^ monocytes **(D)**. Color code: grey, healthy subjects; green, mild COVID-19; orange, moderate COVID-10, red, severe COVID-19. COVID-19 severity was defined according to qCSI, based on respiratory rate, pulse oximetry and oxygen flow rate. Stars denote significance according to ANOVA test among the three COVID groups (mild, moderate, severe), *p < 0.005, **p < 0.001.

T-distributed stochastic neighbor embedding (t-SNE) analysis showed increase of classical (CD14^++^CD16^-^) and intermediate (CD14^++^CD16^+^) monocytes in COVID-19 patients compared to healthy subjects matched for age and sex (Fig. 4A,B). Based on CD16 and CD14 expression, we followed sequential stages of maturation and distinct functions of monocytes shown in Fig. 4C^18,19^. In the pool of intermediate CD14^++^CD16^+^ monocytes the mean of fluorescence intensity of the activation marker CD64 was significantly increased in COVID-19 patients compared to healthy subjects, respectively 599 ± 43 vs 556 ± 39, p = 0.04 (Supplementary Fig. 2, positively associated to the amount of IL-6, (respectively, r-square = 0.42, p = 0.04 and r-square = 0.79, p = 0.04, data not shown). The percentage of CD14^+^-HLA-DR^−^ monocytic-like myeloid derived suppressor cells (mo-MDSCs, Fig. 4E) and their absolute number (Fig. 4F) were significantly increased in severe and moderate disease, as well. Expression of activation marker CD64 on monocytes was negatively associated to the absolute counts of CD8^+^CD45R0^+^ cells, IFN-γ and IL-6 (respectively, r-square = −0.34, p = 0.04; r-square = −0.38, p = 0.04 and r-square = −0.31, p = 0.04) and positively associated to IL-1β (r-square = 0.4, p-value 0.002), as shown in Fig. 1.

The percentage and counts of immune-suppressive monocytes (mo-MDSCs) identified as CD14^+^ HLA-DR^−^ monocytes were higher than normal values in severe COVID-19^+^ patients (Fig. 4D), independently from the amount of IL-6, IL-1β, IL-17A, TGF-β, TNF-α and IFN-γ. As expected, the increase of percentage of CD14^+^ HLA-DR^-^ monocytes was negatively associated to the absolute counts of T-cells (r-square = −0.44, p = 0.04), as shown in Fig. 1.

### Lack of viral clearance at 28 days in not vaccinated is associated with multiple perturbed immunological parameters at hospital admission

One patient admitted with severe COVID-19 died after 12 days from admission. At 28 days from hospital admission, viral clearance, identified as two consecutive negative nasal-pharyngeal swabs, was obtained in 7 patients (4 admitted with mild symptoms and 3 with moderate COVID-19), after a median of 18 days (range 14–28).

**Figure 5:**
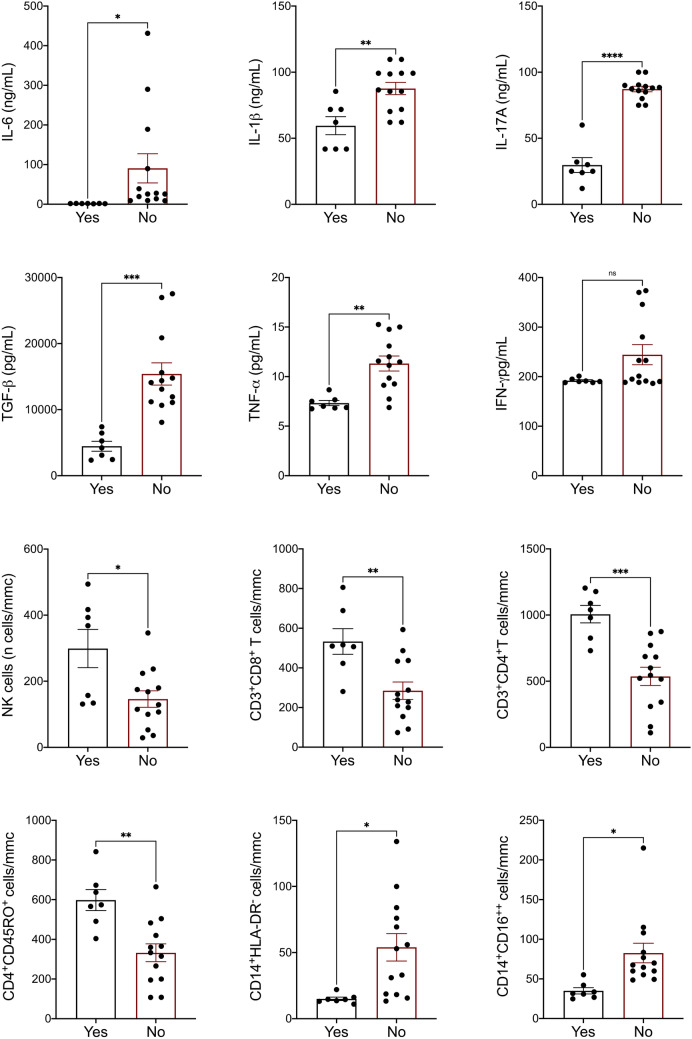
Immune dysregulation at baseline and achievement of viral clearance by 28 days. Amount of circulating cytokines and absolute counts of immune cells in peripheral blood of COVID-19 patienst at hospital admission, distinguished on the achievement of viral clearance by 28 days. Color code: black (patients who achieved viral clearance by 28 days, N = 7); red (patients who did not achieve viral clearance by 28 days, N = 13). Viral clearance was defined as two consecutive negative nasal-pharyngeal swabs. Bars represent mean and standard deviation among the two groups; significance was assessed by T-student test for unpaired samples, *p < 0.005, **p < 0.001, ***p < 0.0001.

In patients who achieved viral clearance by 28 days we found at hospital admission lower amount of IL-6 (p = 0.02), IL-1β (p = 0.03), IL-17A (p < 0.0001), TGF-β (p = 0.003), TNF-α (p = 0.007), IFN-γ (p = 0.01) and absolute counts of effector cells, namely NK-cells (p = 0.01), CD8^+^T cells (p = 0.007), CD4^+^ T-cells (p = 0.0005) and CD4^+^CD45RO^+^ T cells (p = 0.003) as shown in Fig. 5 and Supplementary Table 1. Conversely, absolute counts of immune-suppressive CD14^+^HLA-DR^−^ monocytes (p = 0.01) and inflammatory CD14^+^CD16^++^ monocytes (p = 0.01) were higher at hospital admission in patients who did not achieve viral clearance by 28 days (Fig. 5 and Supplementary Table 1).

In the attempt to build up an immunological classification, we distinguished cases as lower or higher than mean (indicated respectively as *low* or *high* in Supplementary Table 2). Patients defined as *high* based on number of inflammatory monocytes were older than *low* patients; patients defined as *high* based on amount of circulating CD4^+^CD45RO^+^ T-cells received more frequently heparin for their COVID-19 (Supplementary Table 3). When comparing the main outcomes across different immunological classifications, we failed to identify among the subpopulations included in the analysis a biomarker of disease severity, hospitalization/death at 28 days, and time to viral clearance (Supplementary Table 4).

### Severity-dependent alterations of the neutrophil compartment in not vaccinated COVID-19 patients

Despite in our series there was not a significant change in percentage or absolute number of neutrophils between healthy and COVID-19 patients, we detected two main functional abnormalities.

First, median fluorescence intensity (MFI) of the activation marker CD64 on neutrophils was positively associated to the amount of IL-6 (r-square = 0.64, p-value = 0.001), TGF-β (r-square = 0.36, p-value = 0.001), IFN-γ (r-square = 0.5, p-value = 0.002), and IL-17A (r-square = 0.63, p-value = 0.01), at hospital admission, as shown in Fig. 1.

**Figure 6:**
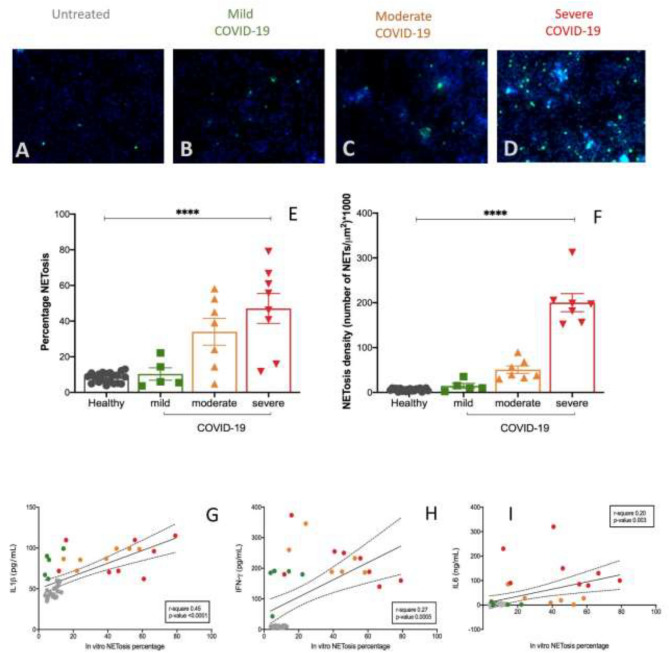
In vitro assessment of NET-osis is associated to clinical severity of COVID-19. High density neutrophils were isolated from three healthy subjects using a density gradient as described in Methods and previously^44–46^; purity was assessed as shown in Supplementary Fig. 1. After isolation, HDNs were maintained in culture with 10% FBS complete RPMI media for 2 h and exposed to sera obtained from healthy or COVID-19 subjects for 3 h. DAPI and cit-H3 staining of DNA revealed the presence of neutrophil extracellular traps (NETs), as shown in panels (**A–D)**. **(E)** percentage of NET-s and **(F)** NET density were quantified and plotted. Bars represent mean and standard deviation. Stars denote ANOVA among the three COVID groups (mild, moderate, severe), *p < 0.005, ** p < 0.001. Correlation among the quantity of percentage of in-vitro NET-osis and amount of IL-1β **(G)**, IFN-γ **(H)** and IL-6 **(I)** is shown. Color code: grey, healthy subjects; green, mild COVID-19; orange, moderate COVID-10, red, severe COVID-19. COVID-19 severity was defined according to qCSI, based on respiratory rate, pulse oximetry and oxygen flow rate.

Second, percentage of NET-osis was progressively higher in moderate and severe COVID-19 patients than in mild disease and controls (as shown in Fig. 6A–D, respectively 25.4 ± 7.5 vs 39.9 ± 9.8 vs 25.5 ± 9.8 vs 10.1 ± 2.1%, Fig. 6E). The NET-osis density, defined as the number of NETs developed in 1 µm^2^ after 3 h of incubation of healthy neutrophils and patients/derived serum, was progressively higher in mild, moderate and severe COVID-19 patients (respectively 86.9 ± 35.2 vs 91.3 ± 32.9 vs 109.3 ± 40.3 cells/µm^2^, Fig. 6F). The percentage of in-vitro induced NET-osis was positively associated to circulating cytokines IL-1β (r-square = 0.45, p-value < 0.0001, Fig. 6G), IFN-γ (r-square = 0.27, p-value = 0.0005, Fig. 6H) and IL-6 (r-square = 0.2, p-value = 0.0003, Fig. 6I). There was no significant difference in percentage of in-vitro NET-osis and NET-osis density in patients who failed to achieve viral clearance at 28 days (data not shown).

### Perturbations of immunome in breakthrough COVID-19 patients

We then compared the quantification and distribution of immune cells of peripheral blood of 12 not vaccinated patients who developed moderate/mild COVID-19 (namely group 1, as described above) with immune profiling of 21 patients (group 2) who developed mild breakthrough COVID-19, despite being fully vaccinated with two doses + one boost dose of vaccine BNT162b2. By study design, diagnosis of COVID-19 was recorded > 4 weeks from the last dose, thus identifying the breakthrough infections after COVID-19 vaccination. General demographics and clinical characteristics are shown in Table 1, last column.

Patients in group 2 had lower frequency of comorbidities and were younger than in group 1, with a median age of 46 years (range 29–63), did not require hospitalization for management of their COVID-19, and achieved viral clearance by 10 days (median 8 days, range 6–10 days). Only half subjects in group-2 achieved a protective titer (> 40 BAU) at one month after two doses of BNT162b2 vaccine, with the median of anti-SARS-CoV-2 antibodies (IgG) titer of 75.2 (range 4–135 BAU).

**Figure 7:**
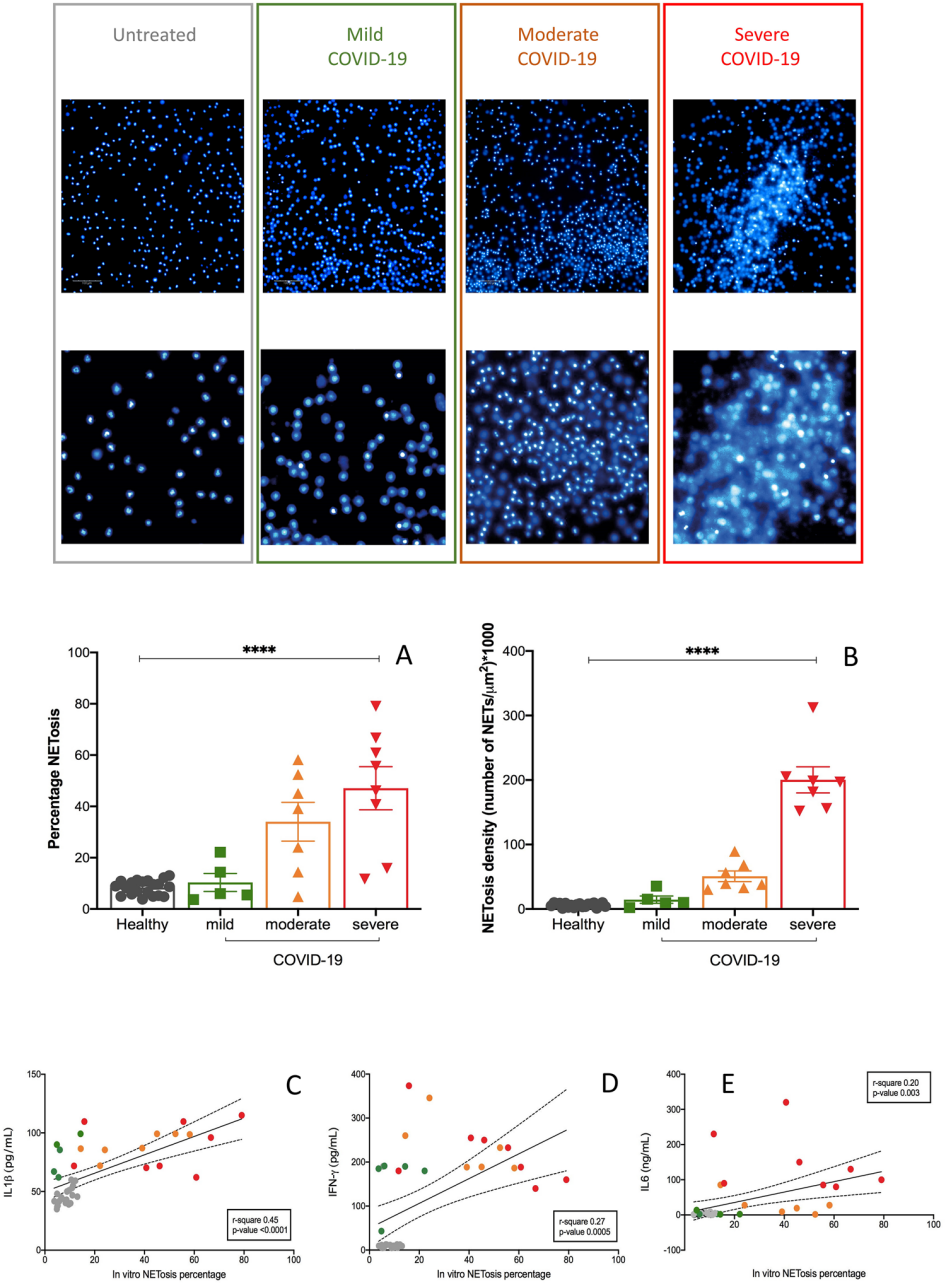
Profiling of immune cells and NETosis in mild-moderate COVID-19 in not vaccinated and in vaccinated patients. High density neutrophils were isolated from three healthy subjects using a density gradient as described in Methods and previously^44–46^; purity was assessed as shown in Supplementary Fig. 1. After isolation, HDNs were maintained in culture with 10% FBS complete RPMI media for 2 h and exposed to sera obtained from COVID-19 subjects, not vaccinated (red squares) or fully vaccinated (blur triangles) for 3 h. Immune profiling was assessed by flow cytometry to evaluate the absolute numbers of CD3^+^CD4^+^ T-cells (**A**), CD3^+^CD4^+^CD45RO^+^ T-cells (**B**), CD3^+^CD8^+^ T-cells (**C**), CD3^+^CD8^+^CD45RO^+^ T-cells (**D**), CD14^+^HLA-DR^-^ monocytes (**E**) and their percentage (**F**). DAPI and cit-H3 staining of DNA revealed the presence of neutrophil extracellular traps (NETs), as shown in the lower panel **(I)**. **(G)** percentage of NET-s and **(H)** NET density was quantified and plotted. Bars represent mean and standard deviation. Stars denote statistical significance when t-Student test for unpaired data was applied, *p < 0.005, **p < 0.001. In experiments using sera from vaccinated patients 5 nM ramipril was added in culture for 1 h before to add patients’sera and NETs detected by immunofluorescence as previously described. A representative image of NETs induced by COVID-19 sera of a vaccinated patient is shown in L panel and its drastic reduction when ramipril was included in the treatment (M panel). Changes in NETs percentage and density have been quantified and plotted respectively in panels N and O. Stars denote statistical significance when t-Student test for paired data was applied, ****p < 0.0001. *NETs* neutrophil extracellular traps, *nM* nanomolar.

As shown in Fig. 7, despite no differences in CD4^+^ and CD8^+^ T-cells absolute counts (panels A–C), compared to group 1, patients in group 2 had higher CD3^+^CD4^+^CD45RO^+^ and CD3^+^CD8^+^CD45RO^+^ circulating cells (respectively, 935 ± vs 487 ± cells/mmc, panel B, p = 0.005 and 563 ± vs 80 ± cells/mmc, p < 0.0001, panel D). No significant differences could be appreciated in absolute counts of neutrophils and monocytes (data not shown), or monocyte subpopulations distribution, absolute counts and percentages of monocytic-like derived suppressor cells CD14^+^-HLA-DR^−^ cells (panels E–F).Sera of breakthrough COVID-19 patients were able to induce more NET-osis in vitro than not vaccinated patients, associated to increased percentage (mean 38.5 ± 4.2 vs 24.1 ± 5.7%, p = 0.04, panel G) and density (mean 227.5 ± 3.2 vs 35.6 ± 7.4, cells/µm^2^, p < 0.0001, panel H).

Since in both group 1 and group 2 hypertension was reported as the most frequent comorbidity and we did not use comorbidity as criteria for matching healthy and COVID-19 patients, we tested if the anti-hypertensive drug more used in group 2, the angiotensin- angiotensin-converting enzyme (ACE) inhibitor ramipril, could affect NET-osis. To this end, we treated neutrophils obtained from healthy, unvaccinated subjects with 5 nM ramipril in vitro for one hour before to add sera obtained from unvaccinated COVID-19 patients for three hours and we found a significant reduction of NET-s percentage and density compared to untreated samples (respectively, mean 10.3 ± 0.9 vs 19.5 ± 1.5%, p < 0.0001, panel N and mean 5.9 ± 0.6 vs 95.7 ± 18.3, cells/µm^2^, p < 0.0001, panel O, t-test for paired samples), as shown in the representative panels L-M of Fig. 7.

## Discussion

In this work we collected information about the composition of immune cells and soluble factors circulating in peripheral blood from symptomatic COVID-19 patients the day after their admission in a dedicated unit. In all patients, the analyses here reported were performed by 24 h after the first positive PCR and not from to the date of first COVID-19 symptom, which represents one of the major limitations of the study, together with the limited number of patients included. Due to variety of symptoms presentation and the inclusion of individuals with mild disease, we preferred to use a uniform starting point for all subjects included in the study.

Not vaccinated patients with moderate or severe disease were older than those with mild disease, even if this difference was not significant. The wide age range reflects the epidemiology of COVID-19 at the study time.

We found significant and global alterations in both T-, B- and myeloid cell compartments that underline the immune system's effort to make up for lymphopenia and loss of naïve T-cells by recruiting switched B-memory cells, besides the cytokine storm and the functional and phenotypic alterations of the compartment of the innate response. Differences of immune composition at baseline could affect the disease evolution since patients with impaired T-cell distribution at hospital admission could not clear viral load by 28 days. Although a positive PCR is not synonymous of active viral development, these results highlight that lower quantity of cytokines and adequate numbers of effector NK and T-cells could contribute to achieve a rapid viral clearance in COVID-19 patients. After infection, the cytokine storm could further amplify immune dysregulation until reverting the protective effects of immune activation in detrimental. We found a positive correlation between intermediate monocytes and IL-6 and reduction of CD4^+^CD45R0^+^cells, suggesting a complex network of interactions between cells of innate and adaptive response.

After an initial weak immune response, SARS-CoV-2 can recruit neutrophils, monocytes and macrophages by inducing a an aggressive and detrimental release of proinflammatory cytokines^20^, associated to peptide specific T-cells^21,22^ and B-cell responses^23,24^, including the activation of memory and naïve B-cell subpopulations^25^, even if in lack of a long-lasting immune response^24^. Our data confirm previous observations of memory B cell loss in COVID-19 patients with acute severe disease^26^, although in our cohort was not possible to make conclusions regarding antigen-specific memory B cells.

Recruitment of CD14^+^CD16^+^ inflammatory monocytes (IM) can play both protective and detrimental roles in viral infections, due their ability to produce Th1-polarizing cytokines, present antigen to CD8+ T cells, promote survival of certain memory CD8^+^ T cell subsets, or promote viral clearance through type I IFN and other cytokines production. As consequence of release of inflammatory cytokines intermediate monocytes can be expanded^27^ and their plastic phenotype followed to monitor the strength of the inflammation status^28^. In some cases, not formally investigated in COVID-19 patients, IMs act as reservoirs for viral replication and support persistence of the viral infection or they can become detrimental for the immune response when they inhibit production of antiviral antibodies through direct interaction with Ag-specific B cells, reducing efficient B cell activation and antibody production^29^. However, IMs do not suppress B cell responses in all settings, due to a different timing of recruitment or persistence of these monocytes in the draining lymph nodes and/or their plastic phenotype, susceptible to the inflammatory environment. Infected monocytes can induce acute inflammatory responses through exerting inappropriate activity and cause cytokine storm (with increased levels of TNF-α, IL-10, and IL-6), which in turn enhances the pathogenicity of the virus, and disease worsening in patients via severe tissue damage^30^.

Several groups reported that severe COVID-19 is characterized by high levels of pro-inflammatory cytokines IL-6 and IL-1β^7,19,31–34^, while IFN responses are blunted, as shown by whole blood transcriptomics^20,35^ and plasma profiling^36^. Our series confirmed a positive correlation between the amount of IFN-γ and IL-17A, associated to the severity of disease as previously reported for MERS-CoV, SARS-CoV and SARS-CoV-2^37,38^, despite we could not test the specific effect of each single cytokine inhibition due to the heterogeneity and small numbers of our series of not vaccinated patients. However, breakthrough infections were mild and not associated to increased amount of C-RP, cytokines or inflamed monocytes expansion. NET-osis in neutrophils can ben induced by IL-6^39^, IL-1 beta^40^ and IFN-gamma^15^, that we fond increased and associated to NET-osis percentage and disease activity.

In not vaccinated patients, we found a positive correlation between NET-s detected in vitro and COVID-19 severity, even if not associated to 28-days viral clearance. Our data point to the critical role for neutrophils in the disease severity and occurrence despite full cycle of vaccination, since the median size of NETs at hospital admission in not vaccinated patients was associated to severity grading, and independent from the amount of circulating cytokines. In severe or persistent viral infections, including COVID-19, neutrophil-mediated alveolar damage leads to interstitial edema, ventilation/perfusion mismatch and respiratory failure. Moreover, the myeloid dysfunction and the disbalance between NETs formation and degradation has been recently addressed as an emerging player in the pathophysiology of inflammation, coagulopathy, organ damage, and thrombosis typical of severe cases COVID-19^41^.

Since hypertension was the most frequent associated comorbidity, we further tested if drugs commonly used for hypertension like ramipril could reduce NET-osis in vitro. Chrysanthopoulou A. et al. that recently disclosed how NETosis can be induced in vitro via an ROS/peptidylarginine deiminase type 4 and autophagy-dependent pathway under control of angiotensin II^42^. In transgenic mice who overexpress ACE, there is increased resistance to bacterial infections through elevated production of superoxide which in turn increases NET formation and cytokine release. In murine neutrophils overexpressing ACE, NETs formation could be reverted to levels of normal mice by exposure to inhibitors of ACE activity^43^. Tang et al. have showed in heart failures murine model that KLF2 regulates neutrophil activation in response to angiotensin II at the molecular level, partly through crosstalk with HIF1 signaling, to control NETosis^44^. Interestingly, in breakthrough infections, we found increased amount of NETs percentage and density, that could be reverted in vitro by exposure to ramipril, an inhibitor of angiotensin converting enzyme (ACE). This finding highlights the complexity of immune-inflammation triggered by SARS-CoV-2 also in vaccinated patients^45^.

In conclusion, our work pointed out some immunological features of not-vaccinated and vaccinated COVID-19 patients and focused on contemporary evaluation of cells of innate and adaptive response, showing that all subsets evaluated were mostly impaired in not-vaccinated COVID-19 patients, while NET-s formation was increased in those patients who developed COVID-19 despite full vaccination cycle completed. The main limitation of our work is the low number of patients involved, despite this is the first report comparing the immune profiling of not-vaccinated and vaccinated subjects who developed COVID-19. Further limitation is that we could not take in account external variables, like polypharmacy, smoking, life habits and so on, neither viral load nor the virus genotype that could affect the basal configuration of the immune system before to be triggered by the infection. It is possible that some of the findings highlighted in this work represent a “core” immunological signature that develops at intermediate-late stages of severe infections or occurrence despite vaccination.

Larger studies able to investigate and predict the complex dynamics of these interactions are required to design effective and suitable treatments.

## Methods

### Study design

We established a research program devoted to collect biological and clinical data in patients monitored for SARS-CoV-2 infection in April 2020, requiring hospitalization, by 7 days from symptoms onset and 24 h from hospital admission. Thus, 20 consecutive patients (aged from 42 to 92 years) affected by symptomatic COVID-19 were admitted at the two largest COVID-19 Units for emerging infectious diseases in Catania, Italy.

Participants meeting eligibility criteria were adults aged 18 or older who have tested positive for SARS-CoV-2 and were at least 10 days away from their first symptoms of infection. Exclusion criteria included the inability to give informed consent and/or an inability to donate plasma or blood transfusion in the past.

At the first day of hospital admission, peripheral blood was collected, and all participants were interviewed to collect information regarding symptoms and their data onset, presence of fever, shortness of breath, sore throat, cough that impacted activity, loss of taste and smell and fatigue. Nasopharyngeal PCR for SARS-CoV-2 were performed at the time of diagnosis and every 7 days or if clinically indicated to assess viral clearance. Clinical records, including laboratory and radiological (chest X-rays or CT scans) findings were collected in a dedicated customized data collection form, reviewed independently by three investigators (M. B., R.M, and L.L.F.) to ensure data accuracy.

The cohort of control subjects included healthy volunteers (age > 18 years), screened by anti-RBD plasma ELISA to confirm negative exposure status.

Written informed consent was collected from every eligible participants and healthy control subject as indicated by the Ethic Committee CATANIA_1 designated for all Units involved in the project (since all Centers share the same Ethic Committee as provided by the institutional declarations available at https://www.policlinicovittorioemanuele.it/comitato-etico-catania-1. All methods and procedures were performed in accordance with the relevant guidelines and regulations provided by the Ethic Committee (https://www.policlinicovittorioemanuele.it/comitato-etico-catania-1).

All samples and data were de-identified following collection, and researchers conducting assays were blinded to clinical data until final comparative analysis.

### Flow cytometric analysis

EDTA anti-coagulated PB samples were stained with the 8-color combination of the monoclonal antibodies: CD11a FITC (25.3), CD18 PE (7E4), CD64 ECD (22), HLA-DR PC5 (Immu-357),CD14PC7(RMO52), CD16 ALEXA-750 (3G8), CD45 Krome Orange (J33), for the evaluation of the monocyte component and with the 9-color combination of the monoclonal antibodies: CD8 FITC (B9.11), CD4 PE(13B8.2), CD3 ECD (UCHT1), CD56 PC5 (N901), CD45RO PC7(UCHL1), CD158b APC (GL183), CD16 ALEXA-750 (3G8), CD45RA Pacific Blue (2H4), CD45 Krome Orange (J33), for the evaluation of T- and Natural Killer (NK) lymphocytes, then incubated for 15 min, at room temperature, in darkness. After this, a red cell lysis procedure was performed, followed by wash procedures. Cell pellet was resuspended in 0.5 ml of PBS. For the phenotypic characterization of the B cells subsets, we used the Dura Clone IM B Cells kit (Beckman Coulter), a reagent panel of 8 monoclonal antibodies: IgD (FITC), CD21 (PE), CD19 (ECD), CD27 (PC7), CD24 (APC), CD38 (APC-750), IgM (Pacific Blue), and CD45 (Krome Orange), according to the manufacturer’s instructions. All antibodies used are obtained from Beckman Coulter. Unstained controls were used to set the flow cytometer photomultiplier tube voltages, and single-color positive controls were used to adjust instrument compensation settings. Data from stained samples were acquired using a Beckman Coulter Navios flow cytometer, equipped with Kaluza software (BD Biosciences) and were analyzed using Kaluza™ Software 2.1 (Becton, Dickinson and Company, Ashland, Oregon, USA, https://www.beckman.it/flow-cytometry/software/kaluza-c), with Infinicyt 2.0 (Cytognos, https://www.cytognos.com) and with R.4.1 (https://www.r-project.org/). Precision Count Beads™ were used to obtain absolute counts of cells acquired by flow cytometer.

t-SNE visualization was generated using Cytobank™ (v.1.0, https://www.beckman.it/flow-cytometry/software/cytobank-enterprise) automatic (t-SNE) learning configuration, with 1000 iterations, a perplexity of 30, learning rate of 3179, exact KNN algorithm, and Barnes-Hut gradient algorithm.

Data were analyzed using *FlowCT*, a semi-automated workflow recently developed for deconvolution of immunophenotypic data and objective reporting on large datasets^46–48^. Briefly, this is a four-step bioinformatic approach including: (1) data assessment (normalization, cleaning); (2) clustering and dimensional reduction through self-organizing map using FlowSOM (version 1.14.1) and UMAP; (3) manual annotation of clusters according to their characteristics markers; and 4) statistical comparisons.

### Serological assays

Serum was obtained from 5 ml peripheral clotted blood after centrifugation at 900 × g and stored in aliquots at − 20 °C. Cytokine levels were measured in serum by using suspension array technology in multiplex using a Milliplex kit for human IL-6, IL-1β, IL-17A, TGF-β, TNF-α, IFN-γ (Millipore Corp, St Charles, Mo) according to the manufacturer’s instructions.

Anti-SARS-CoV-2 ELISA IgG and IgA were detected on using the Euroimmun assays serum samples according to the manufacturer’s instructions. The ratio interpretation was as follows: < 0.8 = negative, ≥ 0.8 to < 1.1 = borderline, ≥ 1.1 = positive*.*

In vaccinated patients, the titer of antibodies developed against the receptor-binding domain of the SARS-CoV-2 spike protein was measured 30 days after the second dose of BNT162b2 vaccine using the SARS-CoV-2 IgG II Quant assay (Abbott, CE marked), by chemiluminescence (CMIA) method, performed on the Abbott Alinity-i platform according to the manufacturer’s instructions.

### Measure of NETotic capacity of COVID-19 serum

Whole blood (20 mL) was collected from 3 healthy volunteers, in vacutainer tubes containing the anticoagulant, potassium EDTA, and diluted 1:1 with dextran 3% for two hours to obtain plasma enriched with white cells. Peripheral blood mononuclear cells (PBMCs) were then isolated by the standard method of density gradient centrifugation using Ficoll-Paque (Pharmacia LKB Biotechnology, NJ, USA). The resulting interphase layer from the gradient was diluted and washed twice with Dulbecco’s phosphate-buffered saline (PBS) (Celbio) to obtain PBMC from the top and neutrophils from the bottom, as previously described^49–51^. Neutrophils with purity and viability of more than 95% were used for further assays (Supplementary Fig. 1). Briefly, the purified neutrophils were allowed to rest in the incubator, at 37 °C, for 30 min, seeded at 10 × 10^4^ cells/mL in a 96-wells plate. As positive control, NET-osis was induced treating cells with the agonist PMA 20 nM for or 5 μL of serum of interest for 120 min at room temperature. Stimulation was stopped by adding 4% paraformaldehyde (PFA) for 30 min at room temperature. Fixed cells were then sequentially stained with Hoechst 33342 (BD Pharmingen, cat#: 561908) and 1.5 μg/mL of antibodies against citrullinated histone H3 (citrulline R2+ R8+ R17; Cat. #5103, Abcam, Cambridge, MA, USA)^40^. Visualization of labelled neutrophils was performed with a high content imager (Operetta, PerkinElmer; 10× objective).

### Statistical analysis

Statistical analyses were performed using GraphPad Prism version 8.0 (GraphPad Software, Inc., San Diego, CA, USA, https://www.graphpad.com). Data were expressed as mean ± the standard deviation or median with interquartile range (IQR) for the quantitative variables, and as numbers and percentages for the categorical variables, where appropriated. Categorical variables were expressed as counts and percentages (%). Groups were compared using the Chi-square or Fisher’s exact test for categorical variables and using the Student’s t test or Mann–Whitney U test for continuous ones. The strength and direction of association between immunological parameters was investigated by applying the Pearson correlation coefficient. A two-sided p value of less than 0.05 was considered statistically significant.

### Ethics approval

The study was approved by the local ethical committee (Comitato etico Catania 1) #CO.TIP. 34/2020/PO 0016693 released on 15 Apr 2020.

## Supplementary Information

Supplementary Information.
