# Supplementary material for: In-vitro NET-osis induced by COVID-19 sera is associated to severe clinical course in not vaccinated patients and immune-dysregulation in breakthrough infection
Source: Sci Rep. 2022 May 4;12:7237. doi: 10.1038/s41598-022-11157-0 (PMC9065667; doi:10.1038/s41598-022-11157-0)
Supplement: Supplementary file 1 — Supplementary Information. [file 41598_2022_11157_MOESM1_ESM.pdf]

Supplementary materials

In-vitro NET-osis induced by COVID-19 sera is associated to severe clinical course in not vaccinated patients and immune-dysregulation in breakthrough infection

by Romano A. et al., 2022

List of supplementary Figures

Supplementary Figure 1 Study design, inclusion criteria and flowchart depicting the overall experimental design of this study.

Supplementary Figure 2 Expression of activation markers CD11A, CD18, CD64 and HLA-DR in non-classical, intermediate and classical monocytes

List of supplementary Tables

Supplementary Table 1 Statistical significance of he correlation matrix and heatmap of Pearson's r correlation coefficients among the multiple variables included in the analysis of COVID-19 patients shown in Figure 1

Supplementary Table 2 Concentration of cytokines and absolute counts of immune cells in peripheral blood in patients who achieved viral clearance by 28 days from hospital admission

Supplementary Table 3 Descriptive statistics of immunological classifications

Supplementary Table 4 Comparison of clinical characteristics and treatments according to immunological classifications

Supplementary Table 5 Main outcomes according to immunological classification

Supplementary Figure 1

Study design, inclusion criteria and flowchart depicting the overall experimental design of this study. Neutrophils were isolated from peripheral blood of healthy donors using a density gradient as described in the methods section, and the proportion of CD66b+ cells was calculated among CD15+ cells by flow cytometry. Three hours later neutrophils were incubated with sera obtained from healthy or COVID-19 subjects to quantify in-vitro NET-osis.

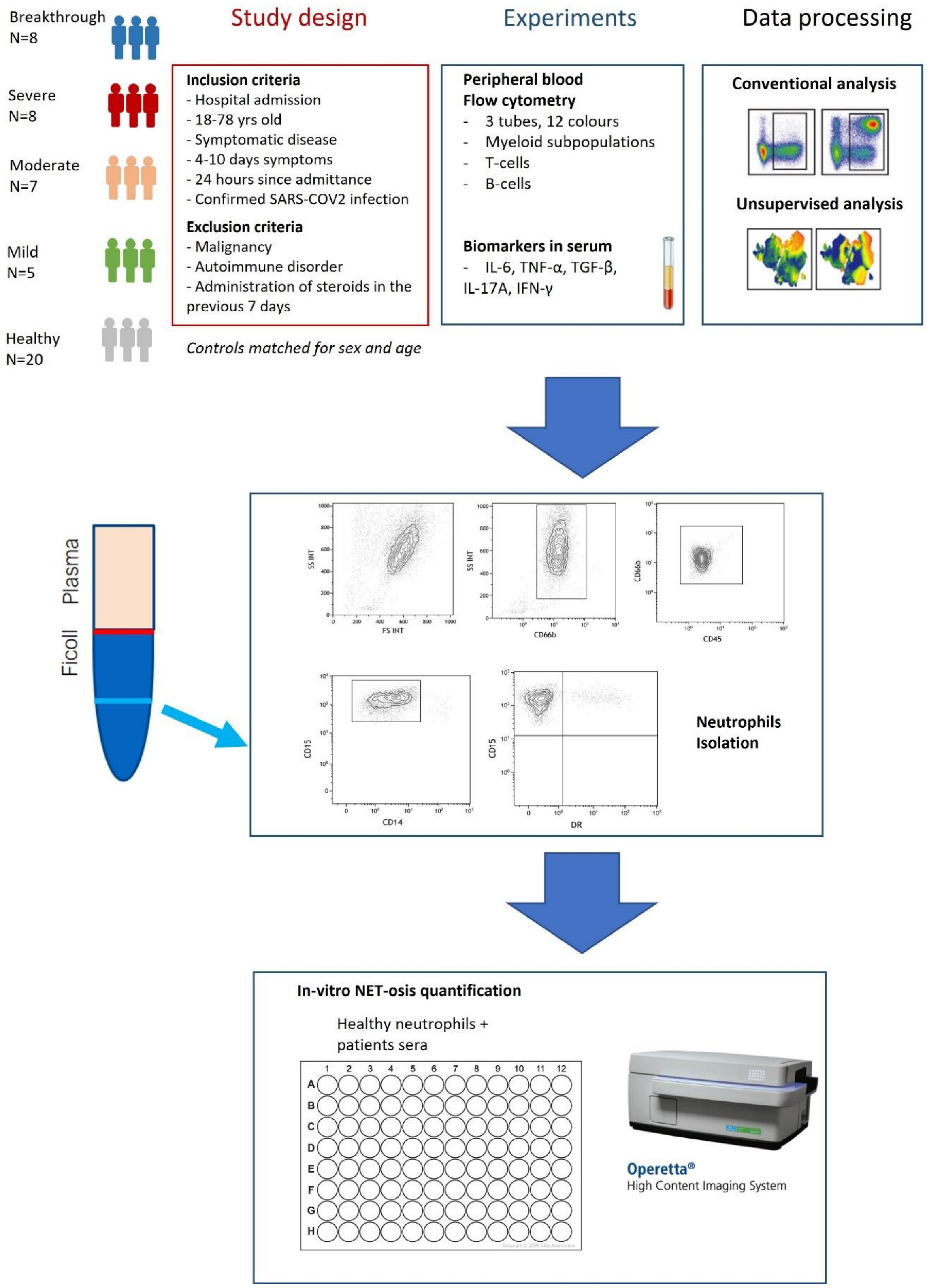

Supplementary Figure 2

Expression of activation markers CD11A, CD18, CD64 and HLA-DR in non-classical, intermediate and classical monocytes

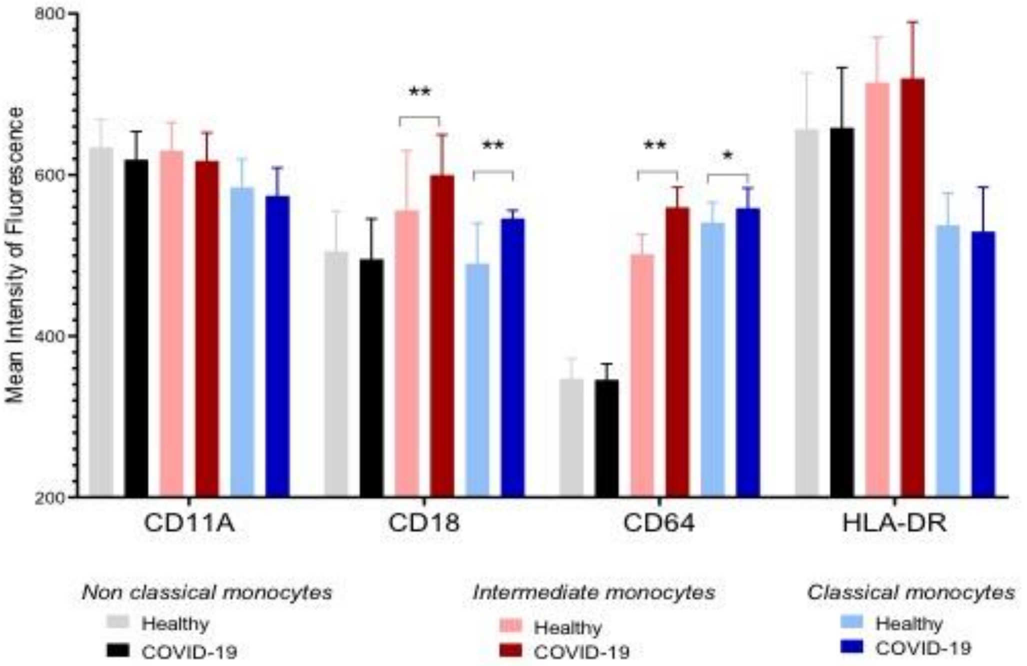

	Non classical monocytes			Intermediate monocytes			Classical monocytes		
	Healthy	COVID	p-value	Healthy	COVID	p-value	Healthy	COVID	p-value
CD11A MFI	633.88	618.77	0.32	630.00	617.41	0.09	584.46	573.92	0.28
CD18 MFI	505.25	495.50	0.08	556.00	599.80	***0.04***	490.09	545.88	***0.03***
CD64 MFI	347.38	345.75	0.32	501.50	559.67	***0.03***	540.78	558.62	***0.04***
HLA-DR MFI	656.50	658.25	0.32	714.67	719.64	0.33	537.26	529.66	0.19

Supplementary Table 1

Statistical significance of he correlation matrix and heatmap of Pearson's r correlation coefficients among the multiple variables included in the analysis of COVID-19 patients shown in Figure 1

	MFI CD64 monocytes	MFI CD11A monocytes	MFI CD18 monocytes	MFI HLA-DR monocytes	MFI CD64 neutrophils	MFI CD16 neutrophils	T/mmc	T4/mmc	T4RA+/mmc	T4RO+/mmc	T8/mmc	mo_MDSC/mmc	T8RA+/mmc	T8RO+/mmc	IL1-β (pg/mL)	TNF-α (pg/mL)	TGF-β (pg/mL)	IFN-γ (pg/mL)	IL-6 (pg/mL)	IL-17A (pg/mL)
MFI CD64 monocytes		0,00	0,231	0,195	0,557	0,380	0,781	0,896	0,227	0,484	0,977	0,139	0,504	0,146	0,079	0,831	0,755	0,094	0,180	0,577
MFI CD11Amonocytes	0,00		0,072	0,02	0,083	0,03	0,956	0,827	0,564	0,636	0,654	0,461	0,537	0,05	0,344	0,415	0,244	0,107	0,080	0,180
MFI CD18monocytes	0,231	0,072		0,235	0,480	0,135	0,346	0,558	0,606	0,210	0,239	0,697	0,299	0,895	0,996	0,576	0,566	0,861	0,684	0,563
MFI HLA-DRmonocytes	0,195	0,02	0,235		0,434	0,03	0,368	0,910	0,173	0,787	0,331	0,610	0,127	0,906	0,442	0,405	0,732	0,899	0,508	0,430
MFI CD64neutrophils	0,557	0,083	0,480	0,434		0,057	0,580	0,795	0,487	0,915	0,637	0,465	0,249	0,02	0,687	0,645	0,121	0,03	0,00	0,00
MFI CD16neutrophils	0,380	0,03	0,135	0,03	0,057		0,160	0,698	0,310	0,156	0,346	0,799	0,513	0,207	0,133	0,647	0,083	0,163	0,217	0,083
T/mmc	0,781	0,956	0,346	0,368	0,580	0,160		0,538	0,01	0,179	0,00	0,082	0,01	0,204	0,970	0,124	0,153	0,507	0,04	0,184
T4/mmc	0,896	0,827	0,558	0,910	0,795	0,698	0,538		0,891	0,261	0,733	0,757	0,921	0,170	0,859	0,570	0,182	0,864	0,731	0,660
T4RA+/mmc	0,227	0,564	0,606	0,173	0,487	0,310	0,01	0,891		0,256	0,071	0,01	0,00	0,234	0,892	0,085	0,05	0,231	0,04	0,115
T4RO+/mmc	0,484	0,636	0,210	0,787	0,915	0,156	0,179	0,261	0,256		0,092	0,03	0,578	0,215	0,135	0,841	0,00	0,880	0,333	0,055
T8/mmc	0,977	0,654	0,239	0,331	0,637	0,346	0,00	0,733	0,071	0,092		0,054	0,065	0,367	0,963	0,831	0,479	0,721	0,04	0,302
mo_MDSC/mmc	0,139	0,461	0,697	0,610	0,465	0,799	0,082	0,757	0,01	0,03	0,054		0,108	0,761	0,405	0,505	0,268	0,498	0,02	0,616
T8RA+/mmc	0,504	0,537	0,299	0,127	0,249	0,513	0,01	0,921	0,00	0,578	0,065	0,108		0,064	0,538	0,02	0,070	0,072	0,02	0,02
T8RO+/mmc	0,146	0,05	0,895	0,906	0,02	0,207	0,204	0,170	0,234	0,215	0,367	0,761	0,064		0,540	0,332	0,00	0,088	0,00	0,00
IL1-β (pg/mL)	0,079	0,344	0,996	0,442	0,687	0,133	0,970	0,859	0,892	0,135	0,963	0,405	0,538	0,540		0,288	0,259	0,644	0,468	0,433
TNF-α (pg/mL)	0,831	0,415	0,576	0,405	0,645	0,647	0,124	0,570	0,085	0,841	0,831	0,505	0,02	0,332	0,288		0,138	0,238	0,244	0,318
TGF-β (pg/mL)	0,755	0,244	0,566	0,732	0,121	0,083	0,153	0,182	0,05	0,00	0,479	0,268	0,070	0,00	0,259	0,138		0,207	0,04	0,00
IFN-γ (pg/mL)	0,094	0,107	0,861	0,899	0,03	0,163	0,507	0,864	0,231	0,880	0,721	0,498	0,072	0,088	0,644	0,238	0,207		0,00	0,088
IL-6 (pg/mL)	0,180	0,080	0,684	0,508	0,00	0,217	0,04	0,731	0,04	0,333	0,04	0,02	0,02	0,00	0,468	0,244	0,04	0,00		0,01
IL-17A (pg/mL)	0,577	0,180	0,563	0,430	0,00	0,083	0,184	0,660	0,115	0,055	0,302	0,616	0,02	0,00	0,433	0,318	0,00	0,088	0,01	

Supplementary Table 2

Concentration of cytokines and absolute counts of immune cells in peripheral blood in patients who achieved viral clearance by 28 days from hospital admission

		Viral clearance by 28 days Yes (N=7)	No (N=13)	p-value[a]
**IL1-β**	Mean (pg/mL)	65.3	86.1	*0.03*
	SD	6.9	6.1	
**TNF-α**	Mean (pg/mL)	7.4	10.9	*0.007*
	SD	0.3	0.8	
**TGF-β**	Mean (pg/mL)	5159	15392	***0.003***
	SD	867	1835	
**IFN-γ**	Mean (pg/mL)	193.1	258.8	***0.01***
	SD	1.5	24.7	
**IL-6**	Mean (pg/mL)	3.8	123.5	***0.02***
	SD	1.1	49.9	
**IL-17A**	Mean (pg/mL)	30.6	89.2	***<0.0001***
	SD	8.2	1.4	
**NK-cells**	Mean (cells/mmc)	299	150	***0.01***
	SD	58	26	
**$CD8^+$ T cells**	Mean (cells/mmc)	533	292	***0.007***
	SD	65	47	
**$CD4^+$ T cells**	Mean (cells/mmc)	1007	532	***0.0005***
	SD	67	75	
**$CD4^+CD45RO^+$ T-cells**	Mean (cells/mmc)	598	343	***0.003***
	SD	53	47	
**$CD14^+HLA\text{-}DR^-$ monocytes**	Mean (cells/mmc)	17	56	***0.01***
	SD	2	12	
**$CD14^+CD16^+$ monocytes**	Mean (cells/mmc)	38	86	***0.01***
	SD	5	15	

[a] based on T-Student tests; p-values <0.05 are indicated in bold font.
Results are reported as mean and standard deviation (SD)

Supplementary Table 3 Descriptive statistics of immunological classifications

Characteristics	Frequency (%)
**Inflammatory monocytes%**	
Low	9 (45%)
High	11 (55%)
**moMDSC%**	
Low	11 (55%)
High	9 (45%)
**CD4/CD45RA%**	
Low	15 (75%)
High	5 (5%)
**CD4/CD45R0%**	
Low	3 (15%)
High	17 (85%)

Supplementary Table 4 Comparison of clinical characteristics and treatments according to immunological classifications

Characteristics	Inflammatory monocytes %			moMDSC%			CD4+CD45RA+%			CD4/CD45RO%		
	Low	High	p-value[a]	Low	High	p-value[a]	Low	High	p-value[a]	Low	High	p-value[a]
**Age, years**	53.0 (13.3)	68.9 (11.9)	***0.011***	62.2 (14.4)	61.2 (15.7)	0.891	64.7 (14.6)	58.8 (11.5)	0.118	50.9 (16.0)	63.4 (14.0)	0.168
**Comorbidities**	77.80%	100%	0.099	100%	77.80%	0.099	85.70%	92.30%	0.639	66.70%	94.10%	0.144
**Severity illness at admission**												
Mild	33.30%	18.20%		18.20%	33.30%		28.60%	23.10%		33.30%	23.50%	
Moderate	22.20%	45.50%	0.522	35.40%	33.30%	0.723	14.30%	46.20%	0.339	0.00%	100.00%	0.375
Severe	44.40%	36.40%		45.50%	33.30%		57.10%	30.80%		66.70%	35.30%	
**Pre-infection administration of drugs**	77.80%	72.70%	0.795	81.80%	66.70%	0.436	85.70%	69.20%	0.417	66.70%	7.60%	0.718
**Post-infection administration of steroids**	66.70%	36.40%	0.178	54.50%	44.40%	0.653	71.40%	38.50%	0.16	66.70%	47.10%	0.531
**Heparin administration**	44.40%	63.60%	0.391	54.50%	55.60%	0.964	28.60%	69.20%	0.081	0.00%	64.70%	***0.038***

[a] based-on Chi-squared or T-Student tests; p-values <0.05 are indicated in bold font
Results are reported as mean (SD) or percentage

Supplementary Table 5 Main outcomes according to immunological classification

Immunological classification		Pneumonia or more severe conditions*	Hospitalized or died at 28 days*	Time To Viral Clearance (days)*
**Inflammatory monocytes %**	**Low**	63.6%	11.1%	23.7 (13.4)
	**High**	77.8%	9.1%	36.4 (15.6)
	**p-value[a]**	0.492	0.999	0.126
**moMDSC%**	**Low**	77.8%	0%	32.4 (10.8)
	**High**	63.6%	22.2%	29.2 (24.3)
	**p-value[a]**	0.492	0.189	0.724
**CD4/CD45RA%**	**Low**	60.0%	13.3%	31.4 (16.8)
	**High**	73.3%	0%	31.3 (14.5)
	**p-value[a]**	0.573	0.999	0.991
**CD4/CD45R0%**	**Low**	70.6%	0%	20.0 (12.5)
	**High**	66.7%	11.8%	34.2 (15.5)
	**p-value[a]**	0.891	0.999	0.170

*based on Chi-squared, Fisher Exact, or T-Student tests; results are reported as mean (SD) or percentage

Supplementary Table 6

Descriptive statistics of the study population at baseline of their COVID-19 diagnosis

Characteristics	Mild-moderate COVID 19 not vaccinated (group 1, N=12)	Breakthrough COVID19 (group 2, N=21)	p-value[a]
**Median age, years (range)**	54 (41-77)	46 (29-63)	0.07
**Males, N (%)**	5 (41)	10 (48)	0.70
**Two or more comorbidities at admission, N (%)**	10 (83)	0 (0)	***<0.0001***
Diabetes	1 (8)	0 (0)	0.19
Hypertension	7 (58)	3 (14)	0.009
Chronic obstructive pulmonary disease	3 (25)	2 (9)	0.22
Hyperlipidemia	7 (58)	4 (19)	***0.02***
**Pre-admission administration of drugs for comorbidities**	7 (58)	0 (0)	0.82
Median Haemoglobin, g/dL (range)	12.8 (10.4-15.1)	14.3 (9.4-15.6)	0.06
Median Absolute white blood cells *10^6/mmc (range)	5.8 (3.6-7.0)	6.6 (3.4-12.3)	0.19
Median Absolute neutrophils *10^6/mmc (range)	3.8 (3.3-5.1)	4.2 (2.8-6.1)	0.13
Median Absolute lymphocytes *10^6/mmc (range)	1.6 (0.9-2.2)	1.8 (0.6-2.8)	0.83
Median Absolute monocytes *10^6/mmc (range)	0.5 (0.3-0.6)	0.4 (0.2-0.6)	0.96
Median Absolute platelets *10^9/mmc (range)	218 (152-260)	242 (89-410)	0.92
**Median LDH, UI/L (range)**	223 (136-300)	156 (120-332)	***0.04***
**Median Reactive C-protein, UI/L (range)**	6.1 (1.4-22.3)	2.3 (1.8-3.9)	***0.01***
**Median IgA, g/dL (range)**	300 (210-368)	290 (240-360)	0.96
**Median IgM, g/dL (range)**	72 (70-123)	96 (85-132)	0.61
**Median IgG, g/dL (range)**	1050 (930-1376)	1086 (870-1400)	0.66
**Median D-dimer, ng/mL (range)**	245 (88-524)	30 (8-16)	***0.02***
**Median anti-SARS-CoV-2 antibodies (IgG) titer, BAU (range)**	0 (NA)	75.2 (4.0-135.0)	NA

[a] based on ANOVA test for the comparison of findings in COVID-19 patients; p-values <0.05 are indicated in bold font. Results are reported as median and interquartile (IQ) range.
